# BUILDING A PROTOCOL FOR LIVE DISCHARGE FROM HOSPICE: UNDERSTANDING THE APPROACHES, CHALLENGES, AND OPPORTUNITIES

**DOI:** 10.1093/geroni/igac059.2515

**Published:** 2022-12-20

**Authors:** Stephanie P Wladkowski, Tracy Schroepfer, Susan Enguidanos

**Affiliations:** Bowling Green State University, Bowling Green, Ohio, United States; University of Wisconsin-Madison, Madison, Wisconsin, United States; University of Southern California, Los Angeles, California, United States

## Abstract

A live discharge from hospice can occur when a patient stabilized under hospice care no longer meets the life expectancy hospice eligibility criteria. In 2019, 278,400 hospice patients across the United States were discharged alive from hospice care, with 18,096 (6.5%) discharged due to being ‘no longer terminally ill.’ For these individuals and their caregivers, the result is a disruption of care continuity and an often burdensome transition. Hospice care improves end-of-life outcomes for some patients, and a live discharge results in lost access to important supportive services and resources, while the patient remains ‘terminal.’ Further, an increased burden is placed on primary caregivers who may be unprepared for this transition. Currently, there is no explicit discharge process available within hospice to guide practitioners in transitioning patients and their caregivers out of hospice care. This study aimed to garner a deeper understanding of current approaches and accompanying challenges to inform the development of an explicit live discharge protocol. Focus group interviews with hospice social workers at four hospice agencies across the U.S. were conducted. Using thematic analysis, four key themes emerged, including the logistical (n=13) and psychosocial (n=9) approaches, the need for clear professional roles during a live discharge (n=12), and specific challenges (n=14), such as needed services and desired discharge timeline to best support the patient-caregiver dyad. Findings demonstrate the complexities of conducting a live discharge, the uniqueness of each hospice agency, and the need for more research to support a standardized and reimbursable discharge process.

